# A recurrent *RYR1* mutation associated with early-onset hypotonia and benign disease course

**DOI:** 10.1186/s40478-021-01254-y

**Published:** 2021-09-17

**Authors:** Valérie Biancalana, John Rendu, Annabelle Chaussenot, Helen Mecili, Eric Bieth, Mélanie Fradin, Sandra Mercier, Maud Michaud, Marie-Christine Nougues, Laurent Pasquier, Sabrina Sacconi, Norma B. Romero, Pascale Marcorelles, François Jérôme Authier, Antoinette Gelot Bernabe, Emmanuelle Uro-Coste, Claude Cances, Bertrand Isidor, Armelle Magot, Marie-Christine Minot-Myhie, Yann Péréon, Julie Perrier-Boeswillwald, Gilles Bretaudeau, Nicolas Dondaine, Alison Bouzenard, Mégane Pizzimenti, Bruno Eymard, Ana Ferreiro, Jocelyn Laporte, Julien Fauré, Johann Böhm

**Affiliations:** 1 Institut de Génétique et de Biologie Moléculaire et Cellulaire (IGBMC), Inserm U 1258, CNRS UMR 7104, Université de Strasbourg, Illkirch, France; 2 Laboratoire de Diagnostic Génétique, Faculté de Médecine, CHRU, Strasbourg, France; 3 Laboratoire de Biochimie et Génétique Moléculaire, Pôle de Biologie, CHU Grenoble Alpes, Grenoble, France; 4 Univ. Grenoble Alpes, Inserm, U1216, CHU Grenoble Alpes, Grenoble Institut Neurosciences, Grenoble, France; 5 Service de Génétique Médicale, Centre de Référence des Maladies Mitochondriales, Hôpital de l’Archet 2, Nice, France; 6 Neurologie Pédiatrique, Centre de Référence des Pathologies Neuromusculaires Nord-Est-Ile de France, Hôpital d’Enfants, CHRU Vandoeuvre-lès-Nancy, France; 7 Service de Génétique Médicale, Hôpital Purpan, CHU Toulouse, Toulouse, France; 8 Service de Génétique Clinique, CHU Rennes, CLAD Ouest, CCNM, Rennes, France; 9 Service de Génétique Médicale, CHU Nantes, Nantes, France; 10 Centre de Référence des Maladies Neuromusculaires Rares AOC, Filnemus, ERN Euro-NMD, Nantes, France; 11 Service de Neurologie, Centre de Référence des Pathologies Neuromusculaires Nord-Est-Ile de France, CHU Central Nancy, Nancy, France; 12 Pediatric Neurology Department, Centre de Référence des Pathologies Neuromusculaires Nord-Est-Ile de France, Armand Trousseau Hospital, APHP, Paris, France; 13 Service de Génétique Clinique, CHU Rennes, Rennes, France; 14 Centre de Référence Maladies Rares CLAD-Ouest, ERN ITHACA, Hôpital Sud, Rennes, France; 15 Peripheral Nervous System and Muscle Department, CHU Nice, Université Côte d’Azur, Nice, France; 16 Neuromuscular Morphology Unit, Myology Institute, GHU Pitié-Salpêtrière, Paris, France; 17 Department of Pathology, Brest University Hospital, Brest, France; 18 Laboratory of Neurosciences of Brest, Faculté de Médecine et des Sciences de la Santé, Université de Bretagne Occidentale, Brest, France; 19 Service d’histologie, Centre de Référence des Pathologies Neuromusculaires Nord-Est-Ile de France, Hôpital Mondor, UnivParis Est Créteil, Créteil, France; 20 Pathology Department, Armand Trousseau Hospital, AP-HP, Paris, France; 21 Institut de Neurobiologie de la Méditerranée, INSERM UMR1249, Marseille, France; 22 Department of Pathology, Toulouse University Hospital, Toulouse, France; 23 AOC (Atlantique-Occitanie-Caraïbe) Reference Centre for Neuromuscular Disorders, CHU, Toulouse, France; 24 Laboratoire d’Explorations Fonctionnelles, CHU Nantes, Nantes, France; 25 Service de Neurologie, CHU Pontchaillou, Rennes, France; 26 Paediatrics department, Centre d’Action Médico-Sociale Précoce, CHU Pontchaillou, Rennes, France; 27 APHP, Centre de Référence des Pathologies Neuromusculaires Nord-Est-Ile de France, Institut de Myologie, GHU Pitié-Salpêtrière, Paris, France; 28 Basic and Translational Myology laboratory, Université de Paris BFA, UMR 8251, CNRS, Paris, France

**Keywords:** Neuromuscular disorder, Congenital myopathy, Calcium, Muscle weakness, Excitation–contraction coupling, Triad

## Abstract

The ryanodine receptor RyR1 is the main sarcoplasmic reticulum Ca^2+^ channel in skeletal muscle and acts as a connecting link between electrical stimulation and Ca^2+^-dependent muscle contraction. Abnormal RyR1 activity compromises normal muscle function and results in various human disorders including malignant hyperthermia, central core disease, and centronuclear myopathy. However, *RYR1* is one of the largest genes of the human genome and accumulates numerous missense variants of uncertain significance (VUS), precluding an efficient molecular diagnosis for many patients and families. Here we describe a recurrent *RYR1* mutation previously classified as VUS, and we provide clinical, histological, and genetic data supporting its pathogenicity. The heterozygous c.12083C>T (p.Ser4028Leu) mutation was found in thirteen patients from nine unrelated congenital myopathy families with consistent clinical presentation, and either segregated with the disease in the dominant families or occurred de novo. The affected individuals essentially manifested neonatal or infancy-onset hypotonia, delayed motor milestones, and a benign disease course differing from classical *RYR1*-related muscle disorders. Muscle biopsies showed unspecific histological and ultrastructural findings, while *RYR1*-typical cores and internal nuclei were seen only in single patients. In conclusion, our data evidence the causality of the *RYR1* c.12083C>T (p.Ser4028Leu) mutation in the development of an atypical congenital myopathy with gradually improving motor function over the first decades of life, and may direct molecular diagnosis for patients with comparable clinical presentation and unspecific histopathological features on the muscle biopsy.

## Introduction

Muscle contraction is a multistep process involving the conversion of an electrical stimulus into mechanical force, and disturbances of this cascade of events can severely impact on muscle physiology and lead to human disorders. The functionality of the excitation–contraction coupling (ECC) machinery essentially relies on the skeletal muscle triad, a specialized membrane complex composed of a deep sarcolemma invagination known as T (transverse)-tubule and two flanking terminal cisternae of the sarcoplasmic reticulum (SR) [12]. ECC is driven by the voltage-gated L-type Ca^2+^ channel DHPR (dihydropyridine receptor) at the T-tubules and the Ca^2+^ channel RyR1 (ryanodine receptor 1) at the SR. Upon membrane depolarization, DHPR undergoes a conformational change and activates RyR1 across the membrane gap to trigger Ca^2+^ release from the SR. The Ca^2+^ ions hence induce the shortening of the contractile units, resulting in the generation of force [10, 30].

Mutations in *RYR1* have been associated with a variety of dominant and recessive pathologies including malignant hyperthermia susceptibility (MHS, OMIM #145,600) [16, 25], King-Denborough syndrome [6], central core disease (CCD, OMIM #117,000) [29, 38], multi-minicore disease (MmD, OMIM #255,320) [26], centronuclear myopathy [36], congenital fiber-type disproportion (CFTD) [5], core-rod myopathy [28], dusty core disease (DuCD) [15], late-onset axial myopathy [24], Samaritan myopathy [3], and exertional myalgia [9]. CCD is the most common *RYR1*-related myopathy, and a significant number of the identified mutations are also associated with an increased risk of MHS [19]. The vast majority of the CCD mutations are heterozygous missense changes mainly affecting conserved amino acids in the C-terminal part of the protein, and functional studies have shown that the mutations either generate a leaky RyR1 channel, or interfere with DHPR binding, and thereby uncouple excitation from contraction [1, 8, 23]. Affected individuals typically present with childhood-onset hypotonia, slowly or non-progressive proximal muscle weakness, facial weakness, joint hypermobility, contractures, and scoliosis [18]. Muscle biopsies from CCD patients display well-delimited areas with reduced oxidative activity and a variable degree of sarcomeric disorganization running along the longitudinal fiber axis as histopathological hallmark [18].

Here we describe a novel and recurrent *RYR1* mutation in nine unrelated congenital myopathy families with unspecific findings on the muscle biopsy, and a consistent clinical picture with unusual disease course differing from classical CCD, MmD, CNM, or CFTD cases.

## Patients and methods

### DNA sampling

Genomic DNA was prepared from peripheral blood by routine procedures from affected and unaffected members of all nine families with written informed consent according to the declaration of Helsinki and its later amendments. DNA storage and usage were IRB-approved (DC-2012-1693). All nine families were from France.

### Molecular diagnosis

DNA samples from family 5 were processed with the SureSelect Human all Exon 50 Mb capture library v5 (Agilent, Santa Clara, USA), and enriched DNA fragments were exome-sequenced on an Illumina HiSeq2500 (Illumina, San Diego, USA). For patient 12 (family 8), the *RYR1* cDNA was amplified and sequenced following reverse transcription of the muscle RNA. All other families were sequenced for a targeted panel of 210 (MYOdiagHTS) or 145 (Myogr_V2019) neuromuscular disorders genes on a NextSeq550 (Illumina).

The exome and panel sequence data were aligned to the GRCh37/hg19 reference genome, and variants were filtered based on the inheritance and their frequency in gnomAD (http://gnomad.broadinstitute.org/) and in our in-house database containing > 1500 exomes, and ranked in accordance with the clinical and histological characteristics of the patients. The potential pathogenic effect of the prioritized variants was predicted using the Alamut software (https://www.interactive-biosoftware.com/alamut-visual/), and the segregation was verified by Sanger sequencing in all families. The identified *RYR1* mutation was numbered according to GenBank NM_000540.3 and NP_000531.2.

To assess a potential splicing effect, skeletal muscle RNA was extracted from frozen muscle samples from patient 12 (family 8) using the Precellys 24 homogenizer (Bertin Technologies, Montigny-le-Bretonneux, France) and reverse transcribed using the SuperScript® III kit (Invitrogen, Carlsbad, USA).

### Muscle morphology

Open muscle biopsies of the vastus lateralis (families 1 and 5), quadriceps (families 2, 4, 8, and 9), or deltoid (families 3, 6, and 7) were performed between age 1 and 66, and the muscle sections underwent histological routine investigations including haematoxylin & eosin (H&E), nicotinamide adenosine dinucleotide-tetrazolium reductase (NADH-TR), Gomori trichrome, and ATPase (pH 9.4). For electron microscopy, the muscle samples were fixed with glutaraldehyde (2.5%, pH 7.4), post fixed with osmium tetroxide (2%), incubated with 5% uranyl acetate, dehydrated in graded series of ethanol, and embedded in epon resin 812.

## Results

### Clinical reports

The thirteen patients described in this study belong to nine unrelated families and most presented with neonatal or infancy-onset hypotonia and delayed motor milestones, followed by a gradual improvement over the first decades of life and a slow functional deterioration at advanced age. An antenatal disease onset with reduced fetal movements in combination with hydramnios and macrosomia was diagnosed in family 3, and the elder affected individuals from family 1 reported a disease onset during childhood, but early clinical data were not available. Families 1 and 2 showed a dominant disease transmission, while the index patients from families 3, 4, 5, 8, and 9 were sporadic cases without ancestral history of a neuromuscular disorder. The patients from families 6 and 7 reported a dominant disease inheritance, but the affected parent either deceased or the contact was lost, precluding molecular segregation analyses. The clinical and histological features of all families are summarized in Table 1.

**Table 1:** Clinical and histological features of patients with *RYR1* c.12083C>T (p.Ser4028Leu) mutation

Family	Patient	Inheritance	Onset	First signs	Age at last examination	Signs at last examination	Age at muscle biopsy	Muscle morphology	Additional signs
1	1	Dominant	Childhood	Elongated face, dysmorphy, proximal muscle weakness	72	Proximal and distal muscle weakness, steppage gait, requires cane, difficulties climbing stairs, VC 40%	66	Type I fiber atrophy, fiber size variability, mitochondrial mispositioning, elongated mitochondria with crystalloid inclusions, lipid droplets	Ventricular hypertrophy
	2		Childhood	Delayed motor milestones	53	Facial weakness, proximal and axial muscle weakness, difficulties climbing stairs, no running, no jumping, Gowers’s sign	-	N.A	High-arched palate, low serum CK
	3		Infancy	Hypotonia, elongated face, delayed motor milestones	14	Proximal and axial muscle weakness, difficulties climbing stairs, no running, no jumping, hypotonia,	-	N.A	High-arched palate, low serum CK
2	4	Dominant	Infancy	Delayed motor milestones	39	Mild axial muscle weakness, myalgia, joint hyperlaxity, difficulties climbing stairs, MH episode	-	N.A	High-arched palate
	5		Neonatal	Severe hypotonia, delayed motor milestones, recurrent infections	3	Axial muscle weakness, hypotonia, joint hyperlaxity, difficulties climbing stairs	1	Type I and II fiber atrophy	Cardiorespiratory arrest at age 4
	6		Neonatal	Severe hypotonia, delayed motor milestones, respiratory distress, recurrent infections	5	Axial muscle weakness, joint hyperlaxity, fatigability, difficulties climbing stairs, Gowers’s sign	4	Type I fiber predominance, fiber size variability	-
3	7	De novo	Antenatal	Hydramnios, fetal macosomia, severe neonatal hypotonia, respiratory distress, elongated face	20	Facial diplegia, ophthalmoplegia, axial and limb girdle weakness, difficulties climbing stairs, no running, no jumping, Gowers’s sign, VC 70%		Type I fiber predominance, lipid droplets	High-arched palate, scoliosis
4	8	De novo	Neonatal	Hypotonia	3	Delayed motor milestones, joint hyperlaxity	1	Type I fiber predominance, rods, lipid droplets, lipofuscin granules	Pes planus
5	9	De novo	Neonatal	Hypotonia, delayed motor milestones	16	Mild ptosis, axial weakness, no running, no jumping, Gowers’s sign, VC 72%	3	Type II fiber atrophy, internal nuclei	Hallux valgus
6	10	Possibly dominant	Infancy	Delayed motor milestones, frequent falls	48	Ophthalmoparesis, axial and distal weakness, myalgia, steppage gait, fatigability, joint hyperlaxity, VC 75%	28	Type I fiber predominance, internal nuclei	High-arched palate, mild scoliosis
7	11	Possibly dominant	Infancy	Delayed motor milestones, elongated face	43	Mild facial weakness, mild ophthalmoparesis, axial and proximal weakness, frequent falls, fatigability, difficulties climbing stairs, myalgia, Gowers’s sign, VC 83%	33	Type I fiber predominance, fiber size variability, mitochondrial mispositioning	High-arched palate, mild scoliosis
8	12	De novo	Neonatal	Hypotonia, delayed motor milestones	15	Mild axial, girdle, and lower limb weakness, fatigability, Gowers’s sign, joint hyperlaxity	3	Type I fiber predominance, fiber size variability, cores	Moderate asthma, scoliosis, pes cavus
9	13	Sporadic	Infancy	Frequent falls, abnormal gait	15	Mild facial and limb girdle weakness, fatigability, difficulties climbing stairs, joint hyperlaxity, VC 65%	9	Fiber size variability, internal nuclei	High-arched palate, pes cavus

NA = not assessed; CK = creatine kinase; VC = vital capacity

Neonatal hypotonia often involved swallowing difficulties necessitating gastrostomy and was accompanied by respiratory distress in patients 6 (family 2) and 7 (family 3), and by recurrent infections in patients 5 and 6 (both family 2). Delayed motor milestones were apparent in all patients with complete medical records. Independent walking was achieved between 18 and 36 months, and commonly came along with frequent falls and an abnormal gait. In all patients, the muscle phenotype stabilized and often improved with motor maturation over the first decades of life. At the last clinical examination, the patients were between 3 and 72 years old, and all were ambulant. The oldest patient (1, family 1) manifested steppage gait, required a cane for walking, and had difficulties climbing stairs. Patients 2, 3 (both family 1), 7 (family 3), and 9 (family 5) were unable to jump or run, climbing stairs was arduous for all affected individuals from families 1, 2, 3, 7, and 9, a positive Gowers’s sign was noted for patients 2 (family 1), 6 (family 2), 7 (family 3), 9 (family 5), 11 (family 7), and 12 (family 8), and an abnormal gait for patient 1 (family 1), 10 (family 6), and 11 (family 7). Muscle weakness was mostly axial, but proximal and distal muscle weakness were also reported in individual cases. Restrictive respiratory involvement with a vital capacity (VC) ranging from of 40% to 83% was noted in patient 1 (family 1), 7 (family 3), 9 (family 5), 10 (family 6), 11 (family 7), and 13 (family 9). Ophthalmoparesis was diagnosed in patient 7 (family 3), 10 (family 6), and 11 (family 7), and ptosis in patient 9 (family 5). Additional clinical signs included high-arched palate (7x), joint hyperlaxity (7x), elongated face (4x), scoliosis (4x), foot deformities (4x), facial diplegia (1x), dysmorphy (1x), and moderate asthma (1x). A cardiac phenotype with ventricular hypertrophy was diagnosed in patient 1 (family 1), and patient 5 (family 2) unexpectedly deceased at the age of 4 from cardiorespiratory arrest. Noteworthy, patient 4 from family 2 experienced an MH episode following surgery.

Whole-body MRI disclosed general muscle atrophy in patient 7 (family 3) and moderate atrophy and fatty infiltrations in the upper and lower limb muscles with particular involvement of the sartorius and the peroneus muscles in patient 10 (family 6). EMG revealed a myopathic pattern in patient 7 (family 3), 9 (family 5), 10 (family 6), and 13 (family 9), and serum creatine kinase (CK) levels were slightly below the normal reference values in patients 2 and 3 (family 1).

### Unspecific histopathological signs on muscle sections

**Fig. 1:**
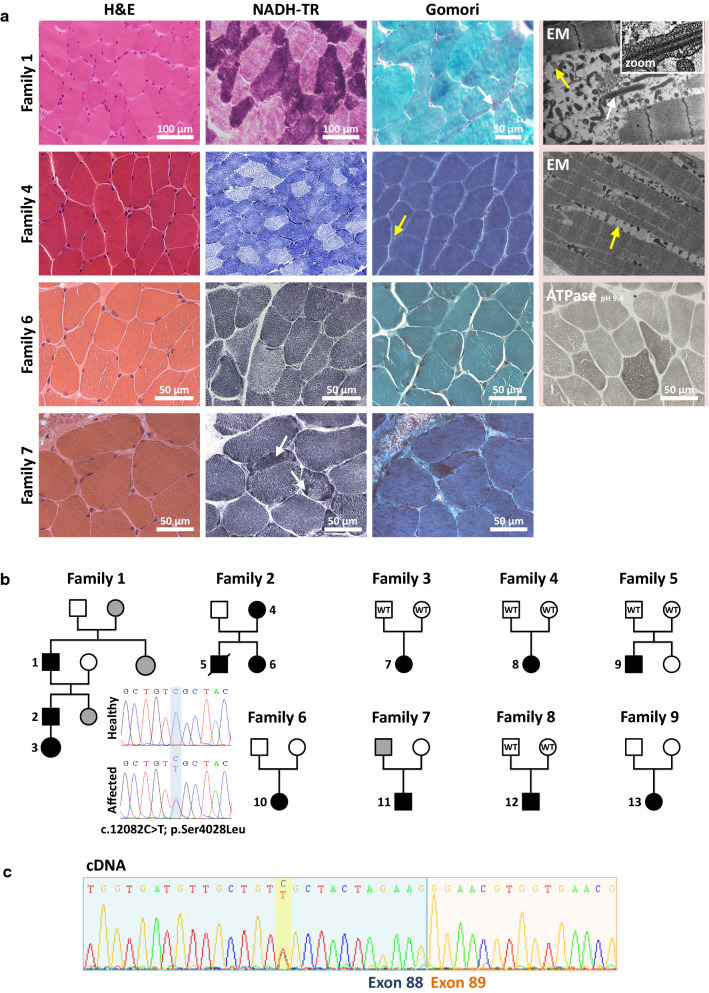
Unspecific histological findings on muscle biopsies. **A** Histological and ultrastructural investigations on muscle sections revealed inconsistent and unspecific findings including fiber size variability on H&E (families 1 and 7), mitochondrial mispositioning on Gomori trichrome and NADH-TR (white arrows, families 1 and 7), atrophy of dark type I fibers on NADH-TR (family 1), predominance of type I fibers on NADH-TR and ATPase (family 6), as well as crystalloid inclusions within mitochondria (white arrow and zoom, family 1) and lipid droplets on EM (yellow arrows, families 1 and 4). Sarcomeric disarray and Z-band streaming were not observed. **B** Pedigrees of the nine families and exemplary electropherograms of healthy and affected individuals indicating the position of the *RYR1* mutation. WT indicates molecular tested healthy individuals, and grey symbols depict reportedly affected individuals without genetic test. **C** Skeletal muscle cDNA sequence of the index patient from family 8 encompassing *RYR1* exons 88 and 89 and excluding a major effect of the mutation on splicing

Patients from all nine families underwent a muscle biopsy, and sections were examined for structural anomalies and abnormal accumulations through a standard panel of histological and histochemical stains (Fig. 1A). The biopsies were taken between age 1 (patient 5, family 2) and age 66 (patient 1, family 1), but morphological analyses did not evidence common and distinct pathological signs or the occurrence of particular features at a specific age. The predominant anomalies included type I fiber predominance (6x), fiber size variability (5x), internal nuclei (3x), mitochondrial mispositioning (2x), type I fiber atrophy (2x), type II fiber atrophy (2x), cores (1x), and rods (1x). Electron microscopy on muscle samples from patients 1 (family 1), 7 (family 3), and 8 (family 4) confirmed mitochondrial mispositioning and aggregation potentially associated with abnormal myofibrillary organization, and additionally uncovered lipid accumulations, lipofuscin granules, and mitochondria containing crystalloid inclusions. COX-negative fibers or signs of muscle fiber degeneration, frequently encountered in mitochondrial myopathies and dystrophies, were not observed. Taken together, the clinical features were similar in all nine families, and the non-specific histological and ultrastructural features on the biopsies were indicative of an undefined congenital myopathy.

### Identification of the RyR1 p.Ser4028Leu mutation

We performed panel sequencing of 210 neuromuscular disorder genes for families 1, 2, 3, 4, and 6, panel sequencing of 145 neuromuscular disorder genes for families 7 and 9, exome sequencing for family 5, and direct cDNA sequencing for family 8. We identified the same heterozygous c.12083C>T transition in *RYR1* exon 88 in all nine families (Fig. 1B), and NGS did not detect other potentially pathogenic variants in known congenital myopathy genes. The *RYR1* mutation segregates with the disease in the dominant families 1 and 2, and occurs de novo in the sporadic cases from families 3, 4, 5, and 8. DNA samples from the parents of the index patient from families 6, 7, and 9 were not available. Although absent from the gnomAD public human variant database (https://gnomad.broadinstitute.org/), c.12083C>T has been referenced by dbSNP (rs794728696) and HGMD (CM157211), where it has been classified as variant of unknown significance (VUS) based on reports of two congenital myopathy patients from China and the Czech Republic with sparse publicly available clinical data [7, 33].

The c.12083C>T mutation involves the substitution of the polar amino acid Serine at position 4028 into the hydrophobic Leucine residue in the central domain of RyR1 (p.Ser4028Leu), and is also predicted to enforce a cryptic acceptor splice site at the 3’ end of exon 88. To explore the potential impact of the mutation on splicing, we extracted RNA from the muscle biopsy from patient 12 (family 8). Sequencing of the reverse-transcribed *RYR1* cDNA did not detect alternative transcripts, and the c.12083C>T mutation appeared at the heterozygous state on the electropherogram, indicating a comparable expression of both alleles in the muscle sample and ruling out a major impact of the mutation on splicing and mRNA stability.

## Discussion

Here we report the identification of a recurrent *RYR1* missense mutation in thirteen patients from nine unrelated families with atypical congenital myopathy associated with a benign disease course. The c.12083C>T (p.Ser4028Leu) mutation was previously classified as VUS, and the present study provides clinical, histological, and genetic data supporting its pathogenicity.

### Potential pathologic impact of the identified ***RYR1*** mutation

The ryanodine receptor RyR1 is an intracellular Ca^2+^ channel mediating skeletal muscle contraction through the rapid release of Ca^2+^ from the sarcoplasmic reticulum to the cytosol [35]. It is composed of more than 5000 amino acids forming a multitude of specialized domains and acting in a highly concerted fashion to enable the transition from open to closed conformation [11, 37]. The central domain encompasses amino acids 3668 to 4251 and serves as a relay station between the cytoplasmic and transmembrane parts to coordinate channel gating [2]. On the resolved protein structure, the Ser4028 residue resides in the first section of the central domain and locates in proximity to the EF-hand motif, implicated in Ca^2+^-dependent regulation of the RyR1 complex [4]. The central domain furthermore constitutes a docking station for diverse allosteric regulators including proteins and small molecules [2]. It is therefore conceivable that the p.Ser4028Leu amino acid substitution directly or indirectly modifies Ca^2+^ sensing or the interaction with RyR1 agonists or antagonists, and thereby interferes with proper channel activity. The differential expression of specific allosteric proteins during muscle development and maintenance may correlate with the disease course in our patients, and provides a potential explanation for the amelioration of the muscle phenotype with age.

### Mutations affecting the RyR1 central domain

To date, almost 700 *RYR1* mutations have been documented and associated with a heterogenous spectrum of human disorders with autosomal dominant or autosomal recessive inheritance [34]. As a general rule, central core disease (CCD) is mainly caused by heterozygous missense mutations often affecting amino acids in the C-terminal pore-forming domain of RyR1, while the less common and phenotypically overlapping multi-minicore disease (MmD), centronuclear myopathy (CNM), and congenital fiber type disproportion (CFTD) arise from recessive missense, splice, and truncating mutations dispersed over the entire gene, and usually involve an earlier disease onset and more severe clinical features compared with CCD [32].

The LOVD database (https://databases.lovd.nl/shared/variants/RYR1/unique) lists 101 variants within or adjacent to *RYR1* exons 75 to 91, encoding the central domain. These variants encompass missense, nonsense, splice, and synonymous single nucleotide substitutions as well as smaller insertions and deletions, and are either classified as pathogenic, benign, or of uncertain significance (VUS). From the 32 heterozygous VUS and pathogenic variants, distinct medical information is only provided for eleven patients, including ten with suspicion of malignant hyperthermia (p.Val3840Ile [17, 31], p.Arg3903Gln [13], p.Ile3916Met [27], p.Gly3938Asp [22], p.Trp3985Arg [21], p.Asp3986Glu [31], p.Thr4081Met [17], p.Arg4136Ser [14], p.Ala4185Thr [22], p.Val4234Leu [13, 14]) and a single patient with muscle weakness and histopathological features of CNM on the biopsy (p.Ser4112Leu [20]). The p.Ser4028Leu missense mutation has been reported in individual congenital myopathy patients from the Czech Republic [33] and China [7], and in accordance with the patients described in the present study, histological investigations on the muscle biopsy from the Chinese patient disclosed nonspecific findings. Taken together, heterozygous missense mutations affecting the RyR1 central domain have primarily been associated with suspicion of malignant hyperthermia, suggesting that p.Ser4028Leu patients may be at risk for MHS. This is supported by the MHS episode in patient 4 from family 2. Noteworthy, variants in *RYR1* exon 88 involving an amino acid change are rare and do not occur at the homozygous state in the healthy population (https://gnomad.broadinstitute.org/), indicating that the encoded section of the RyR1 central domain is particularly intolerant for genetic modifications.

### Concluding remarks

The pathogenicity of the c.12083C>T (p.Ser4028Leu) mutation is supported by several lines of evidence. It segregates with the disease in families with dominant disease transmission, and occurs de novo in sporadic cases. All affected individuals presented with early disease onset and benign progression, and the muscle biopsies displayed a variable picture with unspecific histological signs. Although all nine described families are French, family 1 is of Italian and family 2 of Cambodian origin. A potential impact of the common genetic and ethnic background on the unusual disease presentation can therefore be excluded. This is further sustained by the description of an additional patient from China carrying the same mutation [7], and points to a specific mutational effect of the p.Ser4028Leu mutation on RyR1 function. Overall, our findings provide important insights into the pathogenicity of the RyR1 p.Ser4028Leu mutation previously classified as VUS, and improves the genetic diagnosis for affected patients and families. *RYR1* should also be considered in dominant and sporadic congenital myopathy patients without evocative cores or central nuclei on the muscle biopsy, especially if the patient manifests neonatal or infancy-onset hypotonia improving over time.

## Data Availability

All data generated or analyzed during this study and concerning clinical and histological characteristics and *RYR1* are included in this published article. Other DNA variants identified by panel or exome sequencing are not publicly accessible.
